# 1522–2022: Considerations on the First Description of the Caecal Appendix by Berengario da Carpi in its 500th Anniversary

**DOI:** 10.1007/s00268-022-06688-6

**Published:** 2022-08-08

**Authors:** Michele A. Riva, Marco Ceresoli

**Affiliations:** School of Medicine and Surgery, Milano-Bicocca University, Via Pergolesi 33, 20900 Monza, Italy

The year 2022 marks 500 years from the first description of the vermiform appendix by Jacopo Berengario da Carpi (1466–1530). Although the discovery is nowadays universally attributed to the Italian surgeon and anatomist, few authors correctly indicate the text where this description was first reported. Indeed, some papers still continue to indicate that the first description of the appendix could be found in *Commentaria*, published by Berengario in Bologna in 1521. Actually, the first anatomical description of the appendix was in another text written by the Italian surgeon, *Isagogae Breves*, published one year later, in 1522. This mistake demonstrates that some modern scholars never read the original description by Berengario. This anniversary could appear as a timely opportunity not only to commemorate this discovery, but also to report the original text that hides some underestimated aspects.

## Berengario da Carpi: Biographical Notes

**Fig. 1:**
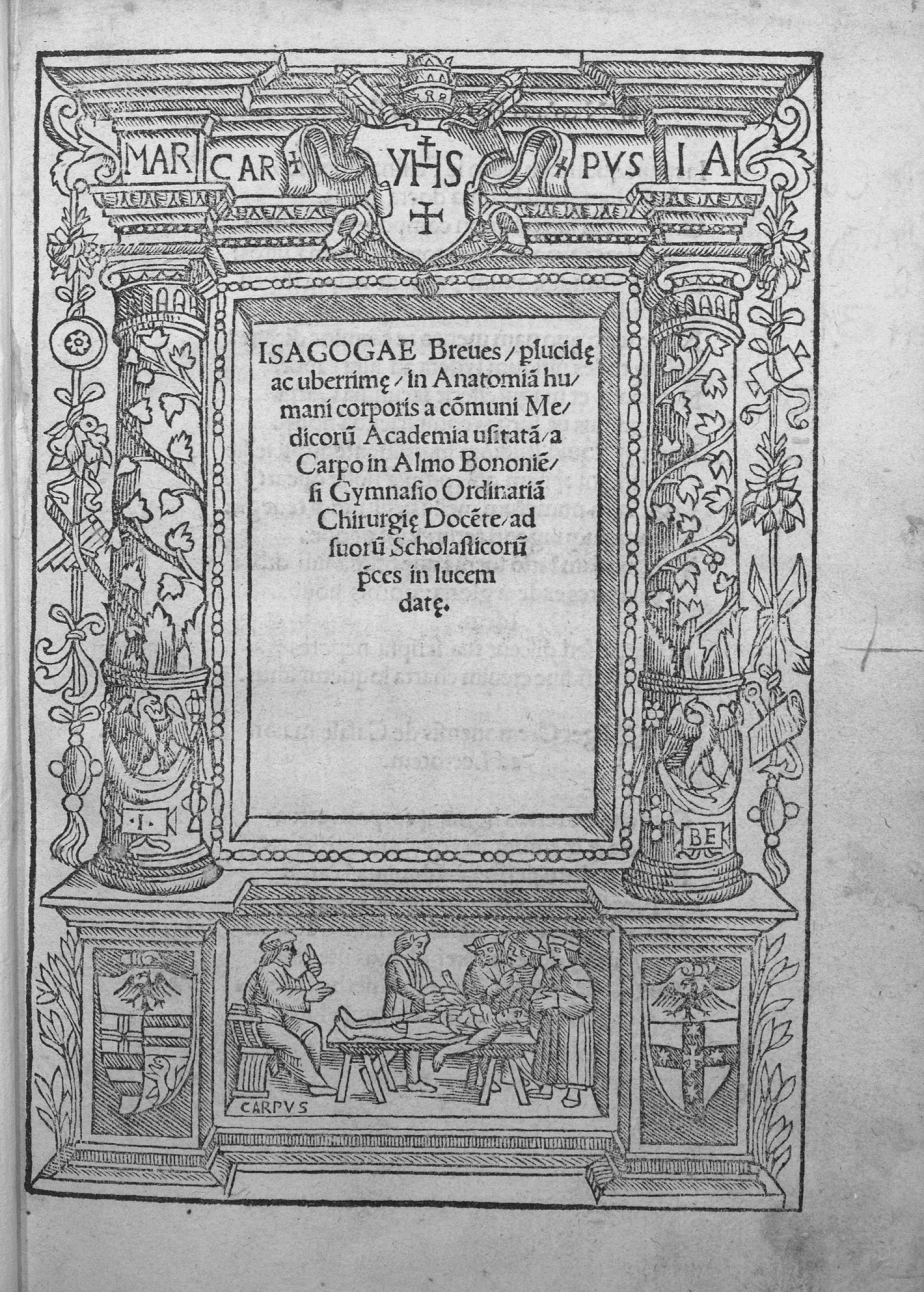
Isagogae Breves by Jacopo Berengario da Carpi. Frontispiece. 1522

Jacopo Berengario da Carpi (Jacopo Barigazzi) was one of the most prominent Italian surgeons of the first half of the sixteenth century. Born in the small town of Carpi, near Bologna, in 1466; his father, Faustino Barigazzi, was a reputable barber-surgeon who initiated his son early to the art of surgery [1]. Jacopo granted his medical degree in 1489 and three years later he was named as Lecturer in Surgery in Bologna, owing his success to his skill in practical medicine and surgery. In 1504, Pope Julius II (1443–1513) granted him Bolognese citizenship, hence confirming his professorship at the University of Bologna, where he taught during nearly a quarter of a century. In that period, he was also appointed as a commissioner of health of the city of Bologna during a plague epidemics. Between 1514 and 1522, Berengario published all his main books and treatises. In particular, in 1521 he completed the *Commentaria cum amplissimis additionibus super Anatomia Mundini* (Commentary on the Anatomy of Mundinus), in which he gives detailed commentaries upon the text *Anothomia,* written by Mondino de’ Liuzzi (ca. 1270–1326) in 1316. The Italian surgeon includes criticisms and emendations to the medieval text by Mondino based on what he had observed during his own dissections [2]. He claimed to have anatomized several hundred bodies. *Commentaria* failed to be a successful publishing edition, because of its large size (527 folio pages) and high costs [3]. So one year later, Berengario published the *Isagogae Breves* (translated in English as ‘A Short Introduction’) (Fig. 1), a compacted little book of only 63 quarto folios, a much shorter and more readable book [4]. This text enjoyed great success, and rapidly went through numerous editions and translations, even abroad. In *Isagogae*, Berengario gives a concise but extremely detailed account of the anatomy of the human body and includes some anatomical illustrations. Indeed, he was also the first anatomist to use illustrations based on direct observations to complement his text, thus proving to be a forerunner of Vesalius. Because of his well-earned fame, Berengario often attended some illustrious patients who lived outside Bologna. Among his patients, the cardinal Pompeo Colonna (1479–1532) could be mentioned; Berengario is believed to have successfully treat a carcinoma which the cardinal was suffering from. In 1517, Lorenzo de’ Medici (1492–1519), Duke of Urbino and nephew of pope Leo X, suffered a skull injury from an harquebus shot and Berengario was asked to come to his bedside. In 1518, the Italian surgeon wrote his *Tractatus de Fractura Calve sive Cranei* (On Fracture of the Calvaria or Cranium) on this case [5], considered as a milestone in the history of neurotraumatology [6, 7].

For unknown reasons, Berengario definitely left Bologna around 1526, moving to Modena and then to Ferrara, where he became court surgeon to the Duke Alfonso I d’Este (1476–1534). In that city, he died in 1530, and was buried there in the local church of Saint Francis.

## The Vermiform Appendix in *Isagogae Breves*

**Fig. 2:**
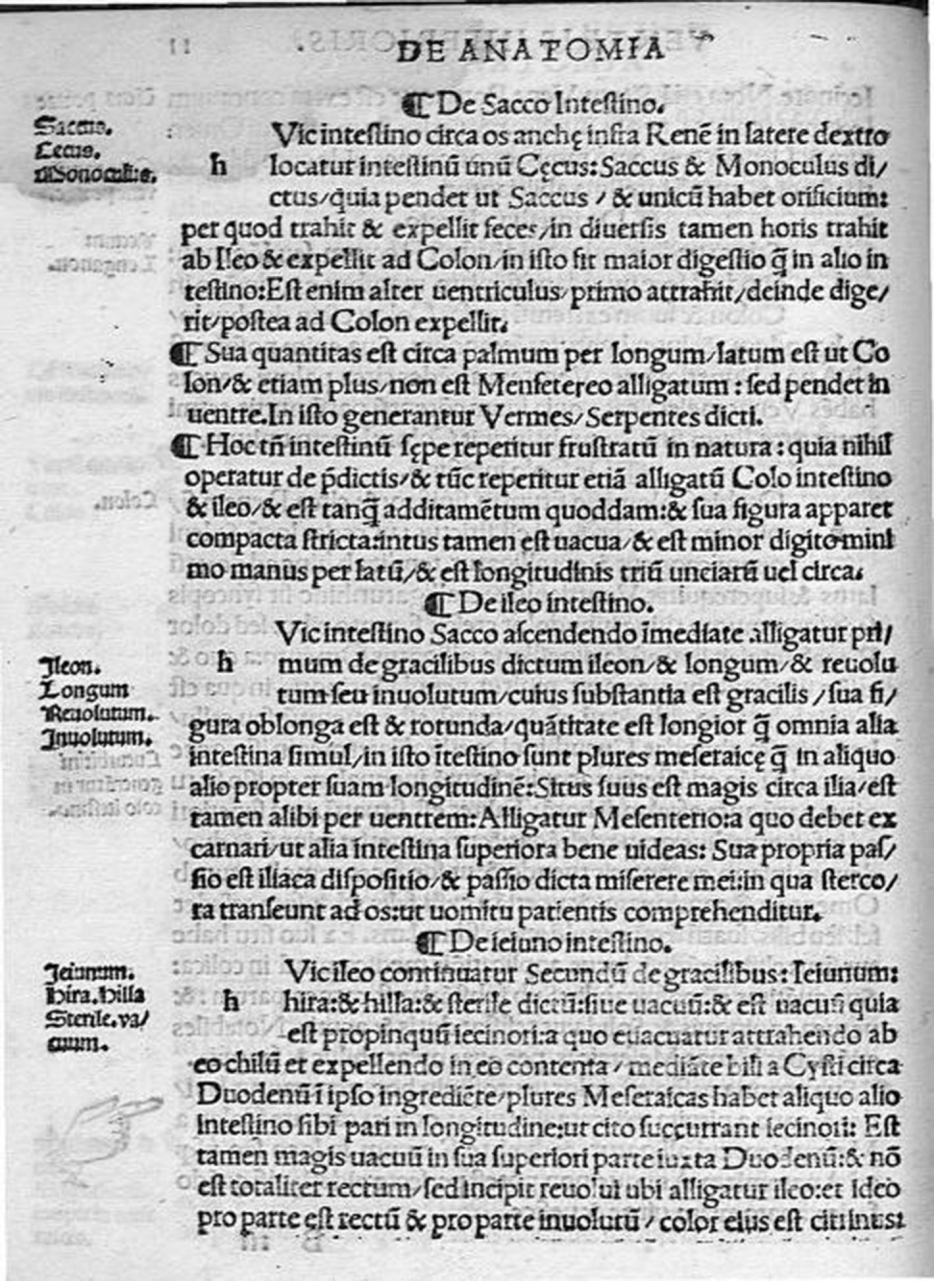
Isagogae Breves by Jacopo Berengario da Carpi. *De Sacco Intestino*. 1522

In *Isagogae Breves*, Berengario divides the human body into three major cavities (abdomen, thorax and head); he begins his description with the lower, proceeding to the middle and upper cavities and finally with the extremities. The description of the vermiform appendix could be found in the first pages of the book and particularly in the section concerning the anatomy of the lower cavity. Between the description of the colon and the ileum intestine, Berengario report an accurate description of the “sack intestine” (*De Sacco Intestino*) (Fig. 2). Here, we reported the translation by the American philologist Levi Robert Lind (1906–2008) in 1959 [3].

“Close to this intestine [colon], near the hipbone below the kidney on the right side, is located the intestine called *cecus*, *saccus*, and *monoculus* because it hangs like a sack and has one orifice through which it draws and expels the feces. At different hours, however, it draws from the ileum and expels to the colon. In this intestine is carried on a greater share of digestion than in any other intestine. For it is another stomach. First it attracts, then digests, and afterward expels to the colon. It is about a span in length. It is as broad as the colon and even more. It is not attached to the mesentery, but hangs in the cavity. In the sack intestine worms called serpents are generated [*in isto generator vermes, serpentes dicti*]. This intestine, however, is often found rendered inoperative [*frustratum*] in nature because it does none of the previously mentioned service. Furthermore, it is found also attached to the colon and to the ileum and is, as it were, a sort of addition [*additamentum*]. Its form appears compactly pressed together. Inside, it is hollow and is less than a little finger in breadth; it is three inches, or nearly that, in length.”

## Discussion

In *Isagogae Breves*, Berengario reported that he found at the end of the caecum a certain “additamentum,” “hollow and is less than a little finger in breadth; it is three inches, or nearly that, in length.” It is probably the first observation of the vermiform appendix.

Berengario described the caecum and the appendix as the same anatomical structure. It is quite interesting to observe that since the first description of the caecum and appendix the interest was focused on their function. In its description, the caecum was reported as the place in which digestion is carried more than in other tract of intestine (“*it attracts, then digests, and afterward expels to the colon*”), up to describe it as a second or another stomach. This description appears to have been influenced by anatomical observations not only on human dissections but also on observation form other mammalians. In fact, the caecum is particularly developed and represented in herbivorous mammalian as horses, and it is the place in which cellulose fibers are fermented and prepared to be assimilated. In this description, it could be observed the influence of Galen’s (ca 129–201) anatomical and physiological descriptions. As known Galen’s study of anatomy largely centered on the dissection of animals, drawing direct comparisons between the physical structures of animals to the human body [8]. So it does not come as a surprise that Berengario believed that the role of cecum in humans was similar to other mammalians. In particular, according to Galen, the functional purpose of the large intestine in humans and the most advanced animals is “to prevent elimination from being a continuous process” [9]. Berengario in the following phrases seems to contest what he previously described *(“this intestine, however, is often found rendered inoperative [frustratum] in nature because it does none of the previously mentioned service”*): this paragraph seems to refers to the human caecum that we know has not digestive functions as in herbivorous.

Berengario also described the presence of worms in the caecum/appendix (“*In the sack intestine worms called serpents are generated [in isto generatur vermes, serpentes dicti]”*): most probably it refers one of the most common helminthiasis caused by the presence of Enterobius Vermicularis, a parasitic nematode of the family of the Oxyuris [10]. Berengario believed that this “serpentes” were generated in this tract of the intestine, similarly to what observed by Galen [11]. In fact, basing on concepts expressed by Aristotle [12], Galen believed that helminths were formed from spontaneous generation (*generatio aequivoca*) in putrefied matter under the effect of heat. He recommended treatment with dietary modification, bloodletting and medicines that were believed to have a cooling and drying effect, in order to return the humoral balance to normal [11].

After the description of the appendix by Berengario several further descriptions were reported: Andreas Vesalius (1514–1564), who published “De Humani Corporis Fabrica” twenty-one years later in 1543, proposed to call this “additamentum” as the *caecum* (“blind”), since the sack intestine has three openings into the ileum, the colon and the appendix itself [13]. In his masterpiece, the first illustration of the appendix can be found in the Fig. 7, Book 5. Vesalius also described it using the words “*vermis in modo convolutus*” (curled in the manner of a worm), so he is credited to liken the appendix to a worm, while the coeval anatomist Eustachius (c. 1510–1574) was the first to use the term “*vermiformis*” in referring to this structure [13]. After the work of Vesalius, in the following two centuries, anatomists gave the name *caecum* to two separate parts of the intestinal tract: the beginning of the colon (also translated in Latin *caput coli*) and the vermiform appendix.

After 500 from its first description nowadays the vermiform appendix still represents a great area of interest in its function and related pathology. Since the first description, the interest has been focused on its function, and curiously, despite 500 years of research and studies, nowadays there are still no clear answers.
